# Examining if being overweight really confers protection against dementia: Sixty-four year follow-up of participants in the Glasgow University alumni cohort study

**DOI:** 10.1186/s12952-016-0062-z

**Published:** 2016-11-02

**Authors:** G. David Batty, Bruna Galobardes, John M. Starr, Mona Jeffreys, George Davey Smith, Tom C. Russ

**Affiliations:** 1 Department of Epidemiology and Public Health, University College, London, UK; 2 Centre for Cognitive Ageing & Cognitive Epidemiology, University of Edinburgh, Edinburgh, UK; 3 Alzheimer Scotland Dementia Research Centre, University of Edinburgh, Edinburgh, UK; 4 School of Social and Community Medicine, University of Bristol, Bristol, UK; 5 Division of Psychiatry, University of Edinburgh, Edinburgh, UK

**Keywords:** Cohort, Dementia, Epidemiology, Life course, Obesity, Overweight, Risk factors

## Abstract

**Background:**

Recent large-scale studies suggest that obesity and overweight may confer *protection* against future dementia. This observation could, however, be generated by reverse causality. That is, weight loss in the incipient phase of dementia ascribed to diminished self-care, including sub-optimal nutrition, would have the effect of generating such an inverse association. One approach to circumventing this problem would be to measure weight in a population which is young enough to be free of the symptoms of dementia which is then followed up for dementia occurrence over many decades.

**Methods:**

In a prospective cohort study, body mass index, and other potential risk factors, were measured in 9547 male university undergraduates (mean age 20.5 years) in 1948–68 who were then linked to national mortality registers.

**Results:**

Of 2537 deaths over a mean of 50.6 years follow up, 140 were ascribed to dementia. There was no association between overweight and future dementia deaths (age-adjusted hazard ratio; 95 % confidence interval: 0.93; 0.49, 1.79).

**Conclusion:**

In this cohort study of former university students, being overweight in youth did not confer protection against later dementia death.

## Introduction

Dementia is a well-documented global health priority and, given projected demographic transitions, substantial increases in the absolute number of people with this disease are anticipated [1]. With current treatments having modest clinical benefit, an improved understanding of the aetiology of dementia is needed if the disorder is to be delayed or prevented. Observations that cerebrovascular pathology commonly co-occurs with Alzheimer’s disease [2], the leading dementia sub-type, has raised the possibility that dementia and cardiovascular disease (CVD) may share similar disease processes. Results from prospective cohort studies suggest that established CVD risk factors when measured in mid- or older-age – smoking, diabetes, physical inactivity, hypercholesterolaemia, and hypertension – are related to dementia risk [3, 4], although these are by no means universal findings [5, 6].

Some reports also suggest that another established CVD risk factor, overweight/obesity, might be associated with an elevated risk of dementia [7]. Other studies, however, including a cohort of 2 million British individuals [8], actually found that being overweight or obese in midlife conferred a *lower* risk of dementia. Results from a recent study accord with these findings [9]. In keeping with these and other discordant results, an expert consensus statement issued by the US National Institutes of Health has indicated that there was insufficient evidence to conclude that overweight/obesity, amongst other modifiable factors, was linked to cognitive decline or dementia [10].

The prolonged preclinical period of many dementias complicates interpretation of findings as to the potential risk factors for this disorder and may explain the controversial overweight/obesity–dementia relationship [11]. That is, the known reduction in weight in the incipient phase of dementia [12, 13] ascribed to diminished self-care, which includes sub-optimal nutrition, would have the effect of generating a potentially spurious inverse association. One approach to addressing this issue of reverse causality is to measure weight in populations which are young enough to be free of the symptoms of dementia who are then followed up for dementia occurrence over many decades. Being unaware of any such data, we report on the long term follow-up for dementia of male undergraduates who had a physical examination which included a measurement of weight, height, and other risk factors at university entry.

## Methods

### Study population

Participants were drawn from the Glasgow Alumni Study which has been described in detail elsewhere [14, 15]. In brief, with the establishment of a student health service at the University of Glasgow (Scotland, UK) in 1947, students were invited to a medical examination on entry.

### Measurement of weight and other risk factors

During an interview and physical examination, a university physician recorded a series of characteristics. Height (inches converted to centimetres) and weight (stones and pounds converted to kilograms) were measured directly. Body mass index (BMI) was calculated using the standard formula (weight[kg]/[height(m)]^2^). Pulse rate (a marker of physical fitness), and systolic and diastolic blood pressure were also recorded. Enquiries were made about father’s occupation (coded according to the Registrar General’s social class schema), amount of physical exertion during recreation (coded as ‘sufficient’, ‘insufficient’), smoking status (nil, slight, moderate, heavy), and alcohol consumption (nil, occasional, regular).

### Ascertainment of dementia death

Individuals enrolling in Glasgow university between 1948 and 1968 were traced using the procedures of the NHS Central Registers to obtain details of emigration, and, for deceased participants, date of death and contributing causes as recorded on death certificates from 1971 onwards. All diagnoses recorded on death certificates were coded according to the International Classification of Diseases (ICD) 9th and 10th revisions. Dementia cases were identified by any mention of codes 290.0 to 290.4, 294.9, 331.0 to 331.2, 331.9 (ICD-9), and codes F00, F01, F03, F09, G30, G31 (ICD-10) [16, 17]. Findings from two studies suggest that using data on dementia death captures the majority of dementia cases. In a UK study, 71.5 % of people with dementia confirmed at a tertiary-referral memory clinic who subsequently died during the next decade had the condition correctly recorded on their death certificates [18] and, in a separate group where multiple sources were used to identify dementia, 83 % of known cases were found using death certificates alone [19].

### Statistical analyses

We excluded women in the cohort (*N* = 2701) as there were too few dementia events (*N* = 21 deaths) in this group to facilitate analyses. In order to focus on a pre-morbid sample, men aged greater than 30 years at university entry were also omitted (*N* = 482). This resulted in a sample of 11,271 men which, after exclusions for the reason of missing data, gave us an analytical sample of 9547. After ascertaining that the proportional hazards assumption had not been violated, we constructed Cox regression models [20] for the association of obesity/overweight and other baseline variables with dementia-related deaths. The timescale was calendar days from examination date with follow-up censored at the date of emigration, death from other causes, or the end of December 2012 (whichever came first). All analyses were conducted using R version 3.2.1.

### Internal and external comparison

To contextualise our data, particularly for weight, we compared baseline characteristics in the Glasgow alumni study with those seen in three contemporary surveys of the Scottish male population (1995, 1998, 2003) [16, 17] in the same baseline age range (16–30 yr). Additionally, to show our data have predictive validity, we also report the associations of overweight/obesity and other risk factors with cardiovascular disease death in the Alumni study. Should known relationships be replicated, this gives us increased confidence in our very novel results for dementia.

## Results

In Table 1 we show the baseline characteristics of the Alumni sample and compare these results with those of men of the same age range from three contemporary Scottish Health Surveys. Levels of CVD risk factors were generally more favourable in the Alumni. This was particularly evident for our principal exposure of interest, BMI: while obesity occurred in 10 % of the present day sample, it was essentially non-existent in the Glasgow Alumni (0.4 %) surveyed up to 55 years earlier. Corresponding values for overweight were 39.9 and 6.8 %. Alumni were also somewhat less likely to smoke and much less likely to drink alcohol but had higher blood pressure. In keeping with a privileged cohort of university students from the era, there was a greater representation of alumni from higher social class backgrounds than in the population-wide Scottish Health Surveys. Comparison of the difference in other baseline characteristics, such as physical inactivity, are complicated by different measurement approaches.

**Table 1:** Comparison of obesity/overweight and other baseline characteristics of male University of Glasgow alumni (1948–68) with male Scottish Health Survey participants (1995, 1998, 2003)

			Glasgow Alumni (*N* = 9547)	Scottish Health Surveys (*N* = 2362)
Age (years)		Mean (SD) Range	20.5 (2.7) 16–30	23.7 (4.5) 16–30
Father’s occupation^a^	I (high)	N (%)	1836 (20.0)	128 (6.6)
	II		3276 (35.7)	301 (15.4)
	III		3401 (37.0)	946 (50.0)
	IV + V		669 (7.3)	577 (30.0)
Physically inactive^b^		N (%)	1049 (12.7)	1346 (57.0)
Body mass index (kg/m^2^)		Mean (SD)	21.6 (2.2)	24.5 (4.1)
Obesity^c^		N (%)	35 (0.4)	219 (10.0)
Overweight		N (%)	647 (6.8)	871 (39.9)
Pulse rate (beats/minute)		Mean (SD)	74.5 (10.3)	70.3 (11.4)
Systolic blood pressure (mmHg)		Mean (SD)	130.7 (13.1)	127.0 (11.1)
Diastolic blood pressure (mmHg)		Mean (SD)	77.2 (8.6)	65.3 (10.4)
Smoker^e^		N (%)	3019 (33.3)	884 (37.9)
Drinks alcohol^f^		N (%)	4483 (54.4)	1605 (75.2)

^a^Categorised according to the UK Registrar General classification of occupations into I (professional), II (intermediate), III (skilled), and IV + V (semi- and unskilled). ^b^Recreation categorised as insufficient, in the examining physician’s opinion (Alumni) or less than five episodes of moderate physical activity per week (SHS). ^c^BMI ≥30 kg/m^2^ for obesity, BMI ≥25 kg/m^2^ for overweight. ^e^Categorised as any or nil. ^f^Categorised as occasional/regular or nil (Alumni) or at least weekly (SHS)

In the analytic sample of 9547 men, an average of 50.6 years follow up gave rise to 2537 (26.6 %) deaths. Of these, 140 study members had dementia recorded on some part of their death certificate and 1157 had mention of CVD but no dementia (42 individuals had both recorded and were included in the dementia analyses but excluded from the CVD analyses). In Table 2 we depict the age-adjusted associations of overweight and other CVD risk factors with dementia and CVD death. In these analyses we collapsed the obese and overweight categories owing to insufficient numbers of dementia deaths (*N* = 2) in the obese group. As anticipated, many of the indices depicted in Table 2 were related to CVD mortality several decades later. This included body mass index where the category of overweight (hazard ratio; 95 % confidence interval: 1.29; 1.05, 1.59) and a standard-deviation-increase in BMI (1.06; 1.00, 1.12) was associated with elevated CVD rates. Other risk factors shown to be related to CVD risk were low childhood socioeconomic status, reduced physical stature, smoking, and higher levels of each component of blood pressure.

**Table 2:** Hazard ratios (95 % confidence intervals) for the association of obesity/overweight and other baseline cardiovascular disease risk factors in relation to dementia and cardiovascular disease^a^ death: University of Glasgow male alumni (*N* = 9547)

		Dementia			Cardiovascular disease		
	N	Deaths	HR (95 % CI)	*P* value	Deaths	HR (95 % CI)	*P* value
Body mass index^b^	9493	140	0.94 (0.80, 1.13)	0.55	1151	1.06 (1.00, 1.12)	0.072
Overweight^c^	9493	140	0.93 (0.49, 1.79)	0.84	1151	1.29 (1.05, 1.59)	0.014
Father occupation^d^	9182	140	1.24 (0.89, 1.73)	0.20	1137	1.13 (1.01, 1.27)	0.040
No physical activity^e^	8240	135	0.50 (0.28, 0.89)	0.018	1090	0.93 (0.78, 1.11)	0.41
Height^b^	9500	140	0.89 (0.76, 1.05)	0.18	1151	1.08 (1.02, 1.14)	0.010
Pulse rate^b^	9184	139	0.96 (0.80, 1.15)	0.67	1141	1.04 (0.98, 1.10)	0.18
Systolic blood pressure^b^	9489	140	0.81 (0.67, 0.98)	0.028	1156	1.15 (1.09, 1.22)	<0.001
Diastolic blood pressure^b^	9468	140	0.84 (0.71, 1.00)	0.054	1153	1.08 (1.02, 1.15)	0.009
Smoker^f^	9068	132	1.42 (1.00, 2.01)	0.047	1088	1.53 (1.36, 1.73)	<0.001
Alcohol consumption^g^	8245	102	1.18 (0.79, 1.76)	0.42	860	0.88 (0.76, 1.01)	0.060

^a^CVD cases were identified by any mention of codes 390–459 for ICD-9 and codes I00-I99 for ICD-10 but no mention of dementia anywhere on the death certificate. ^b^Hazard ratios are age-adjusted and expressed per standard deviation disadvantage (height values were reversed). ^c^BMI ≥25 vs <25 kg/m^2^. ^d^Occupational classes III, IV + V vs I + II (high). ^e^Recreational physical activity categorised as ‘insufficient’ in the examining physician’s opinion. ^f^Any amount of tobacco vs nil. ^g^Occasional/regular drinker vs nil

In the main analyses where we related overweight and other confirmed CVD risk factors to dementia risk, there was little evidence of a gradient. Thus, BMI (one standard-deviation-increase: 0.94; 0.80, 1.13) and overweight (0.93; 0.49, 1.79) were not associated with dementia death at conventional levels of statistical significance. These null relationships were also apparent for father’s occupation, alcohol consumption, height, and pulse rate. Smoking in early adult life was, however, related to an elevated risk of dementia death, while higher levels of both components of blood pressure and physical inactivity were related to lower rates.

## Discussion

The main finding of this study was of no association between overweight in youth and later dementia-related death over a period of up to 64 years. That we found no such link in a group of individuals who would have been free of the symptoms of dementia at weight measurement raises the possibility that the observation of an apparent protective effect of higher BMI against dementia [8, 9] is due to reverse causality. That is, the diminished self-care in people experiencing the early stages of dementia, as manifested by a poor diet, leads to weight loss and a spurious inverse BMI–dementia association. That smoking was associated with an elevated dementia risk appears to support some studies of middle- and older-aged populations [4]. The replication of associations between a range of risk factors and CVD gives us a degree of confidence in our new results for dementia.

The large sample size and long duration of follow up gives us adequate power to identify associations, if they existed. Also, particularly for the era in which these alumni attended university, they would have been among a small, unusually well educated and therefore privileged elite. As such, there would have been very little heterogeneity in educational attainment in these alumni. In aetiological analyses such as our own, this is a distinct advantage: education, known to be related to overweight and dementia, cannot be a confounder in the present dataset when there is no variation in this characteristic.

The study is not of course without its limitations, however. Risk factors were measured only once and levels will have changed in the succeeding decades. Moreover, we analyzed data on men only, so the extent to which our results may be generalized to women is unclear. Lastly, our use of dementia death as our endpoint of interest is somewhat unconventional. As described, however, there is good evidence that the use of death certification captures the majority of dementia cases [18, 19]. As such, we [6, 16, 17, 21, 22], and other groups [9, 23–26], have used dementia death data in other contexts to provide insights into the aetiology of the disorder.

## Conclusion

Overweight was unrelated to dementia deaths in this population of premorbid university alumni. This observation potentially calls into question the previously reported apparent protective role of overweight and obesity against dementia.

## Abbreviations

BMI: body mass index
CVD: cardiovascular disease
ICD: International Classification of Disease

## References

[1] Prince M, Guerchet M, Prina M (2013). Alzheimer’s disease international. Policy brief for heads of government: the global impact of dementia 2013–2050.

[2] Snyder HM, Corriveau RA, Craft S, Faber JE, Greenberg SM, Knopman D (2015). Vascular contributions to cognitive impairment and dementia including Alzheimer’s disease. Alzheimers Dement.

[3] Kivipelto M, Helkala EL, Laakso MP, Hanninen T, Hallikainen M, Alhainen K (2001). Midlife vascular risk factors and Alzheimer’s disease in later life: longitudinal, population based study. BMJ.

[4] Alonso A, Jacobs DR, Menotti A, Nissinen A, Dontas A, Kafatos A (2009). Cardiovascular risk factors and dementia mortality: 40 years of follow-up in the Seven Countries Study. J Neurol Sci.

[5] Fitzpatrick AL, Kuller LH, Lopez OL, Diehr P, O’Meara ES, Longstreth WT (2009). Midlife and late-life obesity and the risk of dementia: cardiovascular health study. Arch Neurol.

[6] Batty GD, Russ TC, Starr JM, Stamatakis E, Kivimaki M (2014). Modifiable cardiovascular disease risk factors as predictors of dementia death: pooling of ten general population-based cohort studies. J Negat Results Biomed.

[7] Kivipelto M, Ngandu T, Fratiglioni L, Viitanen M, Kareholt I, Winblad B (2005). Obesity and vascular risk factors at midlife and the risk of dementia and Alzheimer disease. Arch Neurol.

[8] Qizilbash N, Gregson J, Johnson ME, Pearce N, Douglas I, Wing K (2015). BMI and risk of dementia in two million people over two decades: a retrospective cohort study. Lancet Diab Endocrinol.

[9] Kivimaki M, Singh-Manoux A, Shipley MJ, Elbaz A (2015). Does midlife obesity really lower dementia risk?. Lancet Diab Endocrinol.

[10] Daviglus ML, Bell CC, Berrettini W, Bowen PE, Connolly ES, Cox NJ (2010). National Institutes of Health State-of-the-Science Conference statement: preventing alzheimer disease and cognitive decline. Ann Intern Med.

[11] Sperling RA, Aisen PS, Beckett LA, Bennett DA, Craft S, Fagan AM (2011). Toward defining the preclinical stages of Alzheimer’s disease: recommendations from the National Institute on Aging-Alzheimer’s Association workgroups on diagnostic guidelines for Alzheimer’s disease. Alzheimers Dement.

[12] Stewart R, Masaki K, Xue QL, Peila R, Petrovitch H, White LR (2005). A 32-year prospective study of change in body weight and incident dementia: the Honolulu-Asia Aging Study. Arch Neurol.

[13] Atti AR, Palmer K, Volpato S, Winblad B, De RD, Fratiglioni L (2008). Late-life body mass index and dementia incidence: nine-year follow-up data from the Kungsholmen Project. J Am Geriatr Soc.

[14] McCarron P, Davey Smith G, Okasha M, McEwen J (1999). Life course exposure and later disease: a follow-up study based on medical examinations carried out in Glasgow University (1948–1968). Public Health.

[15] McCarron P, Davey Smith G, Okasha M, McEwen J (2000). Blood pressure in young adulthood and mortality from cardiovascular disease. Lancet.

[16] Russ TC, Stamatakis E, Hamer M, Starr JM, Kivimaki M, Batty GD (2013). Socioeconomic status as a risk factor for dementia death: individual participant meta-analysis of 86 508 men and women from the UK. Br J Psychiatry.

[17] Russ T, Hamer M, Stamatakis E, Starr J, Batty GD (2011). Psychological distress as a risk factor for dementia death. Arch Intern Med.

[18] Russ TC, Batty GD, Starr JM (2012). Cognitive and behavioural predictors of survival in Alzheimer disease: results from a sample of treated patients in a tertiary-referral memory clinic. Int J Geriatr Psychiatry.

[19] Russ TC, Batty GD, Hearnshaw GF, Fenton C, Starr JM (2012). Geographical variation in dementia: systematic review with meta-analysis. Int J Epidemiol.

[20] Cox DR (1972). Regression models and life-tables. J R Stat Soc [Ser B].

[21] Russ TC, Kivimaki M, Starr JM, Stamatakis E, Batty GD (2014). Height in relation to dementia death: individual participant meta-analysis of 18 UK prospective cohort studies. Br J Psychiatry.

[22] Russ TC, Hamer M, Stamatakis E, Starr JM, Batty GD, Kivimaki M (2013). Does the Framingham cardiovascular disease risk score also have predictive utility for dementia death? An individual participant meta-analysis of 11,887 men and women. Atherosclerosis.

[23] Sundstrom A, Westerlund O, Kotyrlo E (2016). Marital status and risk of dementia: a nationwide population-based prospective study from Sweden. BMJ Open.

[24] Rosness TA, Engedal K, Bjertness E, Strand BH (2016). Association between random measured glucose levels in middle and Old Age and risk of dementia-related death. J Am Geriatr Soc.

[25] Koeman T, Schouten LJ, van den Brandt PA, Slottje P, Huss A, Peters S (2015). Occupational exposures and risk of dementia-related mortality in the prospective Netherlands Cohort Study. Am J Ind Med.

[26] Ormstad H, Rosness TA, Bergem AL, Bjertness E, Strand BH (2016). Alcohol consumption in the elderly and risk of dementia related death - a Norwegian prospective study with a 17-year follow-up. Int J Neurosci.

